# On Weak-BCC-Algebras

**DOI:** 10.1155/2013/935097

**Published:** 2013-11-07

**Authors:** Janus Thomys, Xiaohong Zhang

**Affiliations:** ^1^Institute of Mathematics, Wroclaw University of Technology, 50-370 Wroclaw, Poland; ^2^Department of Mathematics, College of Art and Sciences, Shanghai Maritime Univesrity, Shanghai 201306, China

## Abstract

We describe weak-BCC-algebras (also called BZ-algebras) in which the condition (*x*∗*y*)∗*z* = (*x*∗*z*)∗*y* is satisfied only in the case when elements *x*, *y* belong to the same branch. We also characterize ideals, nilradicals, and nilpotent elements of such algebras.

## 1. Introduction

BCK-algebras which are a generalization of the notion of algebra of sets with the set subtraction as the only fundamental nonnullary operation and on the other hand the notion of implication algebra (cf. [1]) were defined by Imai and Iséki in [2]. The class of all BCK-algebras does not form a variety. To prove this fact, Komori introduced in [3] the new class of algebras called BCC-algebras. In view of strong connections with a BIK^+^-logic, BCC-algebras are also called BIK^+^-algebras (cf. [4] or [5]). Nowadays, many mathematicians, especially from China, Japan, and Korea, have been studying various generalizations of BCC-algebras. All these algebras have one distinguished element and satisfy some common identities playing a crucial role in these algebras.

One of very important identities is the identity (*x*∗*y*)∗*z* = (*x*∗*z*)∗*y*. It holds in BCK-algebras and in some generalizations of BCK-algebras, but not in BCC-algebras. BCC-algebras satisfying this identity are BCK-algebras (cf. [6] or [7]). Therefore, it makes sense to consider such BCC-algebras and some of their generalizations for which this identity is satisfied only by elements belonging to some subsets. Such study has been initiated by Dudek in [8].

In this paper, we will study weak-BCC-algebras in which the condition (*x*∗*y*)∗*z* = (*x*∗*z*)∗*y* is satisfied only in the case when elements *x*, *y* belong to the same branch. We describe some endomorphisms of such algebras, ideals, nilradicals, and nilpotent elements.

## 2. Basic Definitions and Facts

**Definition 1**

A weak-BCC-algebra is a system (*G*; ∗, 0) of type (2,0) satisfying the following axioms:

- ((*x*∗*y*)∗(*z*∗*y*))∗(*x*∗*z*) = 0,
- *x*∗*x* = 0,
- *x*∗0 = *x*,
- *x*∗*y* = *y*∗*x* = 0⇒*x* = *y*.

Weak-BCC-algebras are called *BZ-algebras* by many mathematicians, especially from China and Korea (cf. [9] or [10]), but we save the first name because it coincides with the general concept of names presented in the book [11] for algebras of logic.

A weak-BCC-algebra satisfying the identity

(v)  0∗*x* = 0

is called a *BCC-algebra*. A BCC-algebra with the condition

(vi)  (*x*∗(*x*∗*y*))∗*y* = 0

is called a *BCK-algebra*.

One can prove (see [6] or [7]) that a BCC-algebra is a BCK-algebra if and only if it satisfies the identity

(vii)  (*x*∗*y*)∗*z* = (*x*∗*z*)∗*y*.

An algebra (*G*; ∗, 0) of type (2,0) satisfying the axioms (i), (ii), (iii), (iv), and (vi) is called a *BCI-algebra*. A BCI-algebra satisfies also (vii). A weak-BCC-algebra is a BCI-algebra if and only if it satisfies (vii).

Any weak-BCC-algebra can be considered as a partially ordered set. In any weak-BCC-algebra, we can define a natural partial order ⩽ putting

(1) $$\begin{array}{c} x⩽y\Leftrightarrow x*y=0. \end{array}$$

This means that a weak-BCC-algebra can be considered as a partially ordered set with some additional properties.

**Proposition 2**

An algebra (*G*; ∗, 0) of type (2,0) with a relation ⩽ defined by (1) is a weak-BCC-algebra if and only if for all *x*, *y*, *z* ∈ *G* the following conditions are satisfied:

(i′) (*x*∗*y*)∗(*z*∗*y*) ⩽ *x*∗*z*,
(ii′) *x* ⩽ *x*,
(iii′) *x*∗0 = *x*,
(iv′) *x* ⩽ *y  and  y* ⩽ *x  imply  x* = *y*.

From (i′), it follows that in weak-BCC-algebras, implications

(2) $$\begin{array}{c} x⩽y\Rightarrow x*z⩽y*z, \end{array}$$

(3) $$\begin{array}{c} x⩽y\Rightarrow z*y⩽z*x \end{array}$$

are satisfied by all *x*, *y*, *z* ∈ *G*.

A weak-BCC-algebra which is neither BCC-algebra nor BCI-algebra is called *proper*. Proper weak-BCC-algebras have at least four elements (see [12]). But there are only two weak-BCC-algebras of order four which are not isomorphic:

(4) ∗012300022110222220033310

(5) ∗012300022110332220033310

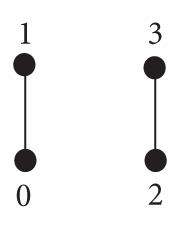
(6)


They are proper, because in both cases (3∗2)∗1 ≠ (3∗1)∗2.

Since two nonisomorphic weak-BCC-algebras may have the same partial order, they cannot be investigated as algebras with the operation induced by partial order. For example, weak-BCC-algebras defined by (4) and (5) have the same partial order but they are not isomorphic.

The methods of construction of weak-BCC-algebras proposed in [12] show that for every *n*⩾4, there exist at least two proper weak-BCC-algebras of order *n* which are not isomorphic.

The set of all minimal (with respect to *⩽*) elements of *G* is denoted by *I*(*G*). Elements belonging to *I*(*G*) are called *initial*.

In the investigation of algebras *G* connected with various types of logics, an important role plays the so-called *Dudek's map φ* : *G* → *G* defined by *φ*(*x*) = 0∗*x*. The main properties of this map in the case of weak-BCC-algebras are collected in the following theorem proved in [13].

**Theorem 3**

Let *G* be a weak-BCC-algebra. Then,

1. *φ* ^2^(*x*) ⩽ *x*,
2. *x* ⩽ *y*⇒*φ*(*x*) = *φ*(*y*),
3. *φ* ^3^(*x*) = *φ*(*x*),
4. *φ* ^2^(*x*∗*y*) = *φ* ^2^(*x*)∗*φ* ^2^(*y*),
5. *φ* ^2^(*x*∗*y*) = *φ*(*y*∗*x*),
6. *φ*(*x*)∗(*y*∗*x*) = *φ*(*y*)

for all *x*, *y* ∈ *G*.

**Theorem 4**

*I*(*G*) = {*a* ∈ *G* : *φ* ^2^(*a*) = *a*}.

The proof of this theorem is given in [14]. Comparing this result with Theorem 3(4), we see that *I*(*G*) is a subalgebra of *G*; that is, it is closed under the operation ∗. In some situations (see Theorem 21), *I*(*G*) is a BCI-algebra.

**Corollary 5**

*I*(*G*) = *φ*(*G*) for any weak-BCC-algebra *G*.

**Proof**

Indeed, if *x* ∈ *φ*(*G*), then *x* = *φ*(*y*) for some *y* ∈ *G*. Thus, by Theorem 3, *φ* ^2^(*x*) = *φ* ^3^(*y*) = *φ*(*y*) = *x*. Hence, *φ* ^2^(*x*) = *x*; that is, *x* ∈ *I*(*G*). So, *φ*(*G*) ⊂ *I*(*G*).

Conversely, for *x* ∈ *I*(*G*), we have *x* = *φ* ^2^(*x*) = *φ*(*φ*(*x*)) = *φ*(*y*), where *y* = *φ*(*x*) ∈ *G*. Thus, *I*(*G*) ⊂ *φ*(*G*), which completes the proof.

This means that an element *a* ∈ *G* is an initial element of a weak-BCC-algebra *G* if and only if it is mentioned in the first row (i.e., in the row corresponding to 0) of the multiplication table of *G*.

Let *G* be a weak-BCC-algebra. For each *a* ∈ *I*(*G*), the set

(7) $$\begin{array}{c} B\left(a\right)=\left\{x\in G:a⩽x\right\} \end{array}$$

is called *a branch of* *G* *initiated by* *a*. A branch containing only one element is called *trivial*. The branch *B*(0) is the greatest BCC-algebra contained in a weak-BCC-algebra *G* ([8]).

According to [1, 15], we say that a subset *A* of a BCK-algebra *G* is an ideal of *G* if (1)  0 ∈ *A*, (2)  *y* ∈ *A* and *x*∗*y* ∈ *A* imply *x* ∈ *A*. If *A* is an ideal, then the relation *θ* defined by

(8) $$\begin{array}{c} x\theta y\Leftrightarrow x*y,\;y*x\in A \end{array}$$

is a congruence on a BCK-algebra *G*. Unfortunately, it is not true for weak-BCC-algebras (cf. [16]). In connection with this fact, Dudek and Zhang introduced in [16] the new concept of ideals. These new ideals are called *BCC-ideals*.

**Definition 6**

A nonempty subset *A* of a weak-BCC-algebra *G* is called a *BCC-ideal* if

1. 0 ∈ *A*,
2. *y* ∈ *A* and (*x*∗*y*)∗*z* ∈ *A* imply *x*∗*z* ∈ *A*.

By putting *z* = 0, we can see that a BCC-ideal is a BCK-ideal. In a BCK-algebra, any ideal is a BCC-ideal, but in BCC-algebras, there are BCC-ideals which are not ideals in the above sense (cf. [16]). It is not difficult to see that *B*(0) is a BCC-ideal of each weak-BCC-algebra.

The equivalence classes of a congruence *θ* defined by (8), where *A* = *B*(0), coincide with branches of *G*; that is, *B*(*a*) = *C*_*a*_ for any *a* ∈ *I*(*G*) (cf. [14]). So,

(9) B(a)∗B(b)={x∗y:x∈B(a),y∈B(b)}=B(a∗b).

In the following part of this paper, we will need those two propositions proved in [14].

**Proposition 7**

Elements *x*, *y* ∈ *G* are in the same branch if and only if *x*∗*y* ∈ *B*(0).

**Proposition 8**

If *x*, *y* ∈ *B*(*a*), then also *x*∗(*x*∗*y*) and *y*∗(*y*∗*x*) are in *B*(*a*).

One of the important classes of weak-BCC-algebras is the class of the so-called *group-like weak-BCC-algebras* called also *antigrouped BZ-algebras* [9], that is, weak-BCC-algebras containing only trivial branches. A special case of such algebras is group-like BCI-algebras described in [17].

From the results proved in [17] (see also [9]), it follows that such weak-BCC-algebras are strongly connected with groups.

**Theorem 9**

An algebra (*G*; ∗, 0) is a group-like weak-BCC-algebra if and only if (*G*; ·, ^−1^, 0), where *x* · *y* = *x*∗(0∗*y*), is a group. Moreover, in this case, *x*∗*y* = *x* · *y* ^−1^.

**Corollary 10**

A group (*G*; ·, ^−1^, 0) is abelian if and only if the corresponding weak-BCC-algebra *G* is a BCI-algebra.

**Corollary 11**

*I*(*G*) is a maximal group-like subalgebra of each weak-BCC-algebra *G*.

## 3. Solid Weak-BCC-Algebras

As it is well known in the investigations of BCI-algebras, the identity (vii) plays a very important role. It is used in the proofs of almost all theorems, but as Dudek noted in his paper [8], many of these theorems can be proved without this identity. Just assume that this identity is fulfilled only by elements belonging to the same branch. In this way, we obtain a new class of weak-BCC-algebras which are called *solid*.

**Definition 12**

A weak-BCC-algebra *G* is called *solid*, if the equation

(vii)  (*x*∗*y*)∗*z* = (*x*∗*z*)∗*y*

is satisfied by all *x*, *y* belonging to the same branch and arbitrary *z* ∈ *G*.

Any BCI-algebra and any BCK-algebra are solid weak-BCC-algebras. A solid weak-BCC-algebra containing only one branch is a BCK-algebra. To see examples of solid weak-BCC-algebras which are not BCI-algebras, one can find them in [8].

**Theorem 13**

Dudek's map *φ* is an endomorphism of each solid weak-BCC-algebra.

**Proof**

Indeed,

(10) φ(x)∗φ(y)=(0∗x)∗(0∗y)=(((x∗y)∗(x∗y))∗x)∗(0∗y)=(((x∗y)∗x)∗(x∗y))∗(0∗y)=(((x∗x)∗y)∗(x∗y))∗(0∗y)=((0∗y)∗(x∗y))∗(0∗y)=((0∗y)∗(0∗y))∗(x∗y)=0∗(x∗y)=φ(x∗y)

for all *x*, *y* ∈ *G*.

**Corollary 14**

*I*(*G*) is a maximal group-like BCI-subalgebra of each solid weak BCC-algebra.

**Proof**

Comparing Corollaries 5 and 11, we see that *I*(*G*) is a maximal group-like subalgebra of each weak BCC-algebra *G*. Thus, by Theorem 9, there exists a group (*I*(*G*); ·, ^−1^, 0) such that *a*∗*b* = *a* · *b* ^−1^ for *a*, *b* ∈ *I*(*G*). Since *G* is solid, *φ* is its endomorphism. Hence, (0∗*a*)∗(0∗*b*) = 0∗(*a*∗*b*) for *a*, *b* ∈ *I*(*G*); that is, *a* ^−1^ · *b* = (*a* · *b* ^−1^)^−1^ = *b* · *a* ^−1^ in the corresponding group. The last is possible only in an abelian group, but in this case, (*a*∗*b*)∗*c* = (*a*∗*c*)∗*b*, which means that *I*(*G*) is a BCI-algebra.

**Definition 15**

For *x*, *y* ∈ *G* and nonnegative integers *n*, we define

(11) $$\begin{array}{c} xy^{0}=x,\;\;x*y^{n+1}=\left(x*y^{n}\right)*y. \end{array}$$

**Theorem 16**

In solid weak-BCC-algebras, the following identity

(12) $$\begin{array}{c} \left(0*x^{k}\right)*\left(0*y^{k}\right)=0*{\left(x*y\right)}^{k} \end{array}$$

is satisfied for each nonnegative integer *k*.

**Proof**

Let *x* ∈ *B*(*a*). Then, by Theorem 3, *a* ⩽ *x* implies 0∗*x* = 0∗*a*. Suppose that 0∗*x* ^*k*^ = 0∗*a* ^*k*^ for some nonnegative integer *k*. Then, also (0∗*a* ^*k*^)∗*x* ⩽ (0∗*a* ^*k*^)∗*a*, by (3). Consequently,

(13) 0∗xk+1=(0∗xk)∗x=(0∗ak)∗x⩽(0∗ak)∗a=0∗ak+1,

which means that 0∗*x* ^*k*+1^ = 0∗*a* ^*k*+1^ because 0∗*a* ^*k*+1^ ∈ *I*(*G*). So, 0∗*a* ^*k*^ = 0∗*x* ^*k*^ is valid for all *x* ∈ *B*(*a*) and each nonnegative integer *k*.

Similarly 0∗*y* ^*k*^ = 0∗*b* ^*k*^ and 0∗(*x*∗*y*)^*k*^ = 0∗(*a*∗*b*)^*k*^ for *y* ∈ *B*(*b*) and nonnegative integer *k*. Thus, a weak-BCC-algebra *G* satisfies the identity (12) if and only if

(14) $$\begin{array}{c} \left(0*a^{k}\right)*\left(0*b^{k}\right)=0*{\left(a*b\right)}^{k} \end{array}$$

holds for *a*, *b* ∈ *I*(*G*). But in view of Corollary 11 and Theorem 9 in the group (*I*(*G*); ·, ^−1^, 0), the last equation can be written in the following form:

(15) $$\begin{array}{c} a^{-k}\cdot b^{k}={\left(a\cdot b^{-1}\right)}^{-k}. \end{array}$$

Since a weak-BCC-algebra *G* is solid, by Corollary 14, *I*(*G*) is a BCI-algebra. So, the group (*I*(*G*); ·, ^−1^, 0) is abelian. Thus, the above equation is valid for all *a*, *b* ∈ *I*(*G*). Hence, (12) is valid for all *x*, *y* ∈ *G* and all nonnegative integers *k*.

**Corollary 17**

The map *φ*_*k*_(*x*) = 0∗*x* ^*k*^ is an endomorphism of each solid weak-BCC-algebra.

**Definition 18**

A weak-BCC-algebra for which *φ*_*k*_ is an endomorphism is called *k*-*strong*. In the case *k* = 1, we say that it is strong.

A solid weak-BCC-algebra is strong for every *k*. The converse statement is not true.

**Example 19**

The weak-BCC-algebra defined by (4) is not solid because (3∗2)∗1 ≠ (3∗1)∗2, but it is strong for every *k*. Indeed, in this weak-BCC-algebra, we have 0∗*x* = 0 for *x* ∈ *B*(0), 0∗*x* = 2 for *x* ∈ *B*(2), and 0∗*x* ^2^ = 0 for all *x* ∈ *G*. So, it is 1-strong and 2-strong. Since in this algebra 0∗*x* ^*k*^ = 0 for even *k*, and 0∗*x* ^*k*^ = 0∗*x* for odd *k*, it is strong for every *k*.

**Example 20**

Direct computations show that the group-like weak-BCC-algebra induced by the symmetric group *S*_3_ (Theorem 9) is *k*-strong for *k* = 5 and *k* = 6 but not for *k* = 1,2, 3,4, 7,8.

**Theorem 21**

A weak-BCC-algebra *G* is strong if and only if *I*(*G*) is a BCI-algebra, that is, if and only if (*I*(*G*); ·, ^−1^, 0) is an abelian group.

**Proof**

Indeed, if *G* is strong, then (0∗*a*)∗(0∗*b*) = 0∗(*a*∗*b*) holds for all *a*, *b* ∈ *I*(*G*). Thus, in the group (*I*(*G*); ·, ^−1^, 0), we have *a* ^−1^ · *b* = (*a* · *b* ^−1^)^−1^ = *b* · *a* ^−1^, which means that the group (*I*(*G*); ·, ^−1^, 0) is abelian. Hence,

(16) (a∗b)∗c=a·b−1·c−1=a·c−1·b−1=(a∗c)∗b

for all *a*, *b*, *c* ∈ *I*(*G*). So, (*I*(*G*); ∗, 0) is a BCI-algebra.

On the other hand, according to Theorem 3, for any *x* ∈ *B*(*a*), *y* ∈ *B*(*b*), we have 0∗*x* = 0∗*a* and 0∗*y* = 0∗*b*. So, if *I*(*G*) is a BCI-algebra, then for any *a*, *b*, *c* ∈ *I*(*G*), we have (*a*∗*b*)∗*c* = (*a*∗*c*)∗*b*. Consequently,

(17) (0∗x)∗(0∗y)=(0∗a)∗(0∗b)=(((a∗b)∗(a∗b))∗a)∗(0∗b)=(((a∗b)∗a)∗(a∗b))∗(0∗b)=(((a∗a)∗b)∗(a∗b))∗(0∗b)=((0∗b)∗(a∗b))∗(0∗b)=((0∗b)∗(0∗b))∗(a∗b)=0∗(a∗b)=0∗(x∗y),

because *x*∗*y* ∈ *B*(*a*∗*b*). This completes the proof.

**Corollary 22**

A strong weak-BCC-algebra is *k*-strong for every *k*.

**Proof**

In a strong weak-BCC-algebra *G*, the group (*I*(*G*); ·, ^−1^, 0) is abelian and 0∗*z* ^*k*^ = 0∗*c* ^*k*^ for every *z* ∈ *B*(*c*). Thus,

(18) (0∗xk)∗(0∗yk)=(0∗ak)∗(0∗bk)=a−k·bk=(a·b−1)−k=0∗(a∗b)k=0∗(x∗y)k

for all *x* ∈ *B*(*a*) and *y* ∈ *B*(*b*).

Example 20 shows that the converse statement is not true; that is, there are weak-BCC-algebras which are strong for some *k* but not for *k* = 1.

**Corollary 23**

A weak-BCC-algebra in which *I*(*G*) is a BCI-algebra is strong for every *k*.

**Corollary 24**

In any strong weak-BCC-algebra, we have

(19) $$\begin{array}{c} 0*\left(0*x^{k}\right)=0*{\left(0*x\right)}^{k} \end{array}$$

for every *x* ∈ *G* and every natural *k*.

## 4. Ideals of Weak-BCC-Algebras

To avoid repetitions, all results formulated in this section will be proved for BCC-ideals. Proofs for ideals are almost identical to proofs for BCC-ideals.

**Theorem 25**

Let *G* be a weak-BCC-algebra. Then, *A* ⊂ *I*(*G*) is an ideal (BCC-ideal) of *I*(*G*) if and only if the set theoretic union of branches *B*(*a*), *a* ∈ *A*, is an ideal (BCC-ideal) of *G*.

**Proof**

Let *S*(*A*) denote the set theoretic union of some branches initiated by elements belonging to *A* ⊂ *I*(*G*); that is,

(20) $$\begin{array}{c} S\left(A\right)=\underset{a\in A}{⋃}B\left(a\right)=\left\{x\in G:x\in B\left(a\right)\text{, }a\in A\right\}. \end{array}$$

By Corollary 11, *I*(*G*) is a weak-BCC-algebra contained in *G*.

If *A* is a BCC-ideal of *I*(*G*), then obviously 0 ∈ *A*. Consequently, 0 ∈ *S*(*A*) because 0 ∈ *B*(0) ⊂ *S*(*A*). Now let *y* ∈ *S*(*A*) and (*x*∗*y*)∗*z* ∈ *S*(*A*) for some *x*, *z* ∈ *G*. Then, *x* ∈ *B*(*a*), *y* ∈ *B*(*b*), *z* ∈ *B*(*c*), and (*x*∗*y*)∗*z* ∈ *B*(*d*) for some *a*, *c* ∈ *I*(*G*) and *b*, *d* ∈ *A*. Thus, (*x*∗*y*)∗*z* ∈ (*B*(*a*)∗*B*(*b*))∗*B*(*c*) = *B*((*a*∗*b*)∗*c*), which means that *B*((*a*∗*b*)∗*c*) = *B*(*d*) since two branches are equal or disjoint. Hence, (*a*∗*b*)∗*c* = *d* ∈ *A*, so *a*∗*c* ∈ *A*. Therefore, *x*∗*z* ∈ *B*(*a*)∗*B*(*c*) = *B*(*a*∗*c*) ⊂ *S*(*A*). This shows that *S*(*A*) is a BCC-ideal of *G*.

Conversely, let *S*(*A*) be a BCC-ideal of *G*. If *a*, (*b*∗*a*)∗*c* ∈ *A* for some *a* ∈ *A* and *b*, *c* ∈ *I*(*G*), then *a* ∈ *B*(*a*) ⊂ *S*(*A*), (*b*∗*a*)∗*c* ∈ *B*((*b*∗*a*)∗*c*) ⊂ *S*(*A*). Hence, *b*∗*c* ∈ *S*(*A*). Since *b*∗*c* ∈ *I*(*G*) and *S*(*A*)∩*I*(*G*) = *A*, the above implies *b*∗*c* ∈ *A*. Thus, *A* is a BCC-ideal of *I*(*G*).

*I*(*G*) is a subalgebra of each weak-BCC-algebra *G*, but it is not an ideal, in general.

**Example 26**

It is easy to check that in the weak-BCC-algebra *G* defined by

(21) ∗0ab000baa0bbbb0

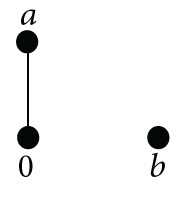
(22)
*I*(*G*) = {0, *b*} is not an ideal because *a*∗*b* = *b* ∈ *I*(*G*), but *a* ∉ *I*(*G*).

The above example suggests the following.

**Theorem 27**

If *I*(*G*) is a proper ideal or a proper BCC-ideal of a weak-BCC-algebra *G*, then *G* has at least two nontrivial branches.

**Proof**

Since {0} ≠ *I*(*G*) ≠ *G*, at least one branch of *G* is not trivial. Suppose that only *B*(*b*) has more than one element. Then, for any 0 ≠ *a* ∈ *I*(*G*) and *x* ∈ *B*(*b*), *x* ≠ *b*, we have *x*∗*a* ∈ *B*(*b*)∗*B*(*a*) = *B*(*b*∗*a*). But, by Corollary 11, *I*(*G*) is a maximal group-like subalgebra contained in *G*. Thus, *b*∗*a* ∈ *I*(*G*) and *b*∗*a* ≠ *b*, because in the case *b*∗*a* = *b* in the corresponding group (*G*; ·, ^−1^, 0), we obtain *b* = *b* · *a* which is impossible for *a* ≠ 0. Therefore, *B*(*b*∗*a*) ≠ *B*(*b*) and *B*(*b*∗*a*) has only one element. So, *x*∗*a* = *b*∗*a*. Hence, *x*∗*a* ∈ *I*(*G*), which according to the assumption on *I*(*G*) implies *x* ∈ *I*(*G*). The obtained contradiction shows that *I*(*G*) cannot be an ideal of *G*. Consequently, it cannot be a BCC-ideal, too.

**Definition 28**

A nonempty subset *A* of a weak-BCC-algebra *G* is called an (*m*, *n*)-*fold p*-*ideal* of *G* if it contains 0 and

(23) $$\begin{array}{c} \left(x*z^{m}\right)*\left(y*z^{n}\right),\;y\in A\Rightarrow x\in A. \end{array}$$

An (*n*, *n*)-fold *p*-ideal is called an *n*-*fold p*-*ideal*. Since (0,0)-fold *p*-ideals coincide with BCK-ideals, we will consider (*m*, *n*)-fold *p*-ideals only for *m*⩾1 and *n*⩾1. Moreover, it will be assumed that *m* ≠ *n* + 1 because for *m* = *n* + 1 we have (*x*∗*x* ^*n*+1^)∗(0∗*x* ^*n*^) = (0∗*x* ^*n*^)∗(0∗*x* ^*n*^) = 0 ∈ *A*, which implies *x* ∈ *A*. So, *A* = *G* for every (*n* + 1, *n*)-fold *p*-ideal *A* of *G*. Note, that the concept of (1,1)-fold *p*-ideals coincides with the concept of *p*-ideals studied in BCI-algebras (see e.g., [18] or [19]).

**Example 29**

It is easy to see that in the weak-BCC-algebra defined by (4), the set *A* = {0,1} is an *n*-fold *p*-ideal for every *n*⩾1. It is not an (*m*, *n*)-fold *p*-ideal, where *m* is odd and *n* is even because in this case (2∗2^*m*^)∗(0∗2^*n*^) ∈ *A* and 0 ∈ *A*, but 2 ∉ *A*.

Putting *z* = 0 in (23), we see that each (*m*, *n*)-fold *p*-ideal of a weak-BCC-algebra is an ideal. The converse statement is not true since, as it follows from Theorem 30 proved below, each (*m*, *n*)-fold ideal contains the branch *B*(0) which for BCC-ideals is not true.

**Theorem 30**

Any (*m*, *n*)-fold *p*-ideal contains *B*(0).

**Proof**

Let *A* be an (*m*, *n*)-fold *p*-ideal of a weak-BCC-algebra *G*. Since for every *x* ∈ *B*(0) from 0 ⩽ *x* it follows that 0∗*x* = 0, we have

(24) $$\begin{array}{c} \left(x*x^{m}\right)*\left(0*x^{n}\right)=\left(0*x^{m-1}\right)*\left(0*x^{n}\right)=0\in A, \end{array}$$

which, according to (23), gives *x* ∈ *A*. Thus, *B*(0)⊆*A*.

**Corollary 31**

An (*m*, *n*)-fold *p*-ideal *A* together with an element *x* ∈ *A* contains whole branch containing this element.

**Proof**

Let *x* ∈ *A* and *y* be an arbitrary element from the branch *B*(*a*) containing *x*. Then, according to Proposition 7, we have *y*∗*x* ∈ *B*(0) ⊂ *A*. Since *A* is also an ideal, the last implies *y* ∈ *A*. Thus, *B*(*a*) ⊂ *A*.

**Corollary 32**

For any *n*-fold *p*-ideal *A* from *x* ⩽ *y* and *x* ∈ *A*, it follows that *y* ∈ *A*.

**Theorem 33**

A nonempty subset *A* of a solid weak-BCC-algebra *G* is its (*m*, *n*)-fold *p*-ideal if and only if

- *I*(*A*) is an (*m*, *n*)-fold *p*-ideal of *I*(*G*),
- *A* = ⋃{*B*(*a*) : *a* ∈ *I*(*A*)}.

**Proof**

Let *A* be an (*m*, *n*)-fold *p*-ideal of *G*. Then, clearly *I*(*A*) = *A*∩*I*(*G*) ≠ *∅* is an (*m*, *n*)-fold *p*-ideal of *I*(*G*). By Corollary 31, *A* is the set theoretic union of all branches *B*(*a*) such that *a* ∈ *I*(*A*). So, any (*m*, *n*)-fold *p*-ideal *A* satisfies the above two conditions.

Suppose now that a nonempty subset *A* of *G* satisfies these two conditions. Let *x*, *y*, *z* ∈ *G*. If *x* ∈ *B*(*a*), *y* ∈ *B*(*b*), *z* ∈ *B*(*c*), and *y*, (*x*∗*z* ^*m*^)∗(*y*∗*z* ^*n*^) ∈ *A*, then (*x*∗*z* ^*m*^)∗(*y*∗*z* ^*n*^) ∈ *B*((*a*∗*c* ^*m*^)∗(*b*∗*c* ^*n*^)), which, by (*b*), implies *b*, (*a*∗*c* ^*m*^)∗(*b*∗*c* ^*n*^) ∈ *I*(*A*). This, by (*a*), gives *a* ∈ *I*(*A*). So, *B*(*a*) ⊂ *A*. Hence, *x* ∈ *A*.

Note that in some situations, the converse of Theorem 30 is true.

**Theorem 34**

An ideal *A* of a weak-BCC-algebra *G* is its *n*-fold *p*-ideal if and only if *B*(0) ⊂ *A*.

**Proof**

By Theorem 30, any *n*-fold *p*-ideal contains *B*(0). On the other hand, if *A* is an ideal of *G* and *B*(0) ⊂ *A*, then from *y* ∈ *A* and (*x*∗*z* ^*n*^)∗(*y*∗*z* ^*n*^) ∈ *A*, by (i′), it follows that

(25) (x∗zn)∗(y∗zn)⩽(x∗zn−1)∗(y∗zn−1)⩽(x∗zn−2)∗(y∗zn−2)⩽⋯⩽x∗y,

so (*x*∗*z* ^*n*^)∗(*y*∗*z* ^*n*^) and *x*∗*y*, as comparable elements, are in the same branch. Hence, (*x*∗*y*)∗((*x*∗*z* ^*n*^)∗(*y*∗*z* ^*n*^)) ∈ *B*(0) ⊂ *A*, by Proposition 7. Since (*x*∗*z* ^*n*^)∗(*y*∗*z* ^*n*^) ∈ *A* and *A* is a BCC-ideal (or a BCK-ideal), (*x*∗*y*)∗((*x*∗*z* ^*n*^)∗(*y*∗*z* ^*n*^)) ∈ *A* implies *x*∗*y* ∈ *A*. Consequently, *x* ∈ *A*. So, *A* is an *n*-fold *p*-ideal.

**Corollary 35**

Any ideal containing an *n*-fold *p*-ideal is also an *n*-fold *p*-ideal.

**Proof**

Suppose that an ideal *B* contains some *n*-fold *p*-ideal *A*. Then, *B*(0) ⊂ *A* ⊂ *B*, which completes the proof.

**Corollary 36**

An ideal *A* of a weak-BCC-algebra *G* is its *n*-fold *p*-ideal if and only if the implication

(26) $$\begin{array}{c} \left(x*z^{n}\right)*\left(y*z^{n}\right)\in A\Rightarrow x*y\in A \end{array}$$

is valid for all *x*, *y*, *z* ∈ *G*.

**Proof**

Let *A* be an *n*-fold *p*-ideal of *G*. Since (*x*∗*z* ^*n*^)∗(*y*∗*z* ^*n*^) ⩽ *x*∗*y*, from (*x*∗*z* ^*n*^)∗(*y*∗*z* ^*n*^) ∈ *A* and by Corollary 32, we obtain *x*∗*y* ∈ *A*. So, any *n*-fold *p*-ideal satisfies this implication.

The converse statement is obvious.

**Theorem 37**

An *n*-fold *p*-ideal is a *k*-fold *p*-ideal for any *k* ⩽ *n*.

**Proof**

Similarly, as in the previous proof, we have

(27) (x∗zn)∗(y∗zn)⩽(x∗zn−1)∗(y∗zn−1)⩽⋯⩽(x∗zk)∗(y∗zk)

for every 1 ⩽ *k* ⩽ *n*. Thus, (*x*∗*z* ^*n*^)∗(*y*∗*z* ^*n*^) and (*x*∗*z* ^*k*^)∗(*y*∗*z* ^*k*^) are in the same branch. Hence, if *A* is an *n*-fold *p*-ideal and (*x*∗*z* ^*k*^)∗(*y*∗*z* ^*k*^) ∈ *A*, then, by Corollary 31, also (*x*∗*z* ^*n*^)∗(*y*∗*z* ^*n*^) ∈ *A*. This, together with *y* ∈ *A*, implies *x* ∈ *A*. Therefore, *A* is a *k*-fold ideal.

**Theorem 38**

*B*(0) is the smallest *n*-fold *p*-ideal of each weak-BCC-algebra.

**Proof**

Obviously, 0 ∈ *B*(0). If *y* ∈ *B*(0), then 0 ⩽ *y*, 0∗*z* ^*n*^ ⩽ *y*∗*z* ^*n*^ and

(28) (x∗zn)∗(y∗zn)⩽(x∗zn)∗(0∗zn)⩽(x∗zn−1)∗(0∗zn−1)⩽⋯⩽x∗0=x.

Thus, (*x*∗*z* ^*n*^)∗(*y*∗*z* ^*n*^) ⩽ *x*. Since (*x*∗*z* ^*n*^)∗(*y*∗*z* ^*n*^) ∈ *B*(0) means 0 ⩽ (*x*∗*z* ^*n*^)∗(*y*∗*z* ^*n*^), from the above, we obtain 0 ⩽ *x*. So, *x* ∈ *B*(0). Hence, *B*(0) is an *n*-fold *p*-ideal. By Theorem 30, it is the smallest *n*-fold *p*-ideal of each weak-BCC-algebra.

**Theorem 39**

Let *G* be a weak-BCC-algebra. If *I*(*G*) has *k* elements and *k* divides |*m* − *n*|, then *B*(0) is an (*m*, *n*)-fold *p*-ideal of *G*.

**Proof**

By Corollary 11, *I*(*G*) is a group-like subalgebra of *G*. Hence, if *I*(*G*) has *k* elements, then in the group (*I*(*G*); ·, ^−1^, 0) connected with *I*(*G*) (Theorem 9), we have *b* ^*ks*^ = 0 for every *b* ∈ *I*(*G*) and any integer *s*.

At first, we consider the case *m*⩾*n*. If (*x*∗*z* ^*m*^)∗(*y*∗*z* ^*n*^) ∈ *B*(0) for some *x* ∈ *B*(*a*), *y* ∈ *B*(0), *z* ∈ *B*(*c*), then, by (i′), we have (*x*∗*z* ^*m*^)∗(*y*∗*z* ^*n*^)⩽(*x*∗*z* ^*m*−*n*^)∗*y*. Hence, (*x*∗*z* ^*m*−*n*^)∗*y* and (*x*∗*z* ^*m*^)∗(*y*∗*z* ^*n*^), as comparable elements, are in the same branch. Consequently, ((*x*∗*z* ^*m*−*n*^)∗*y*)∗((*x*∗*z* ^*m*^)∗(*y*∗*z* ^*n*^)) ∈ *B*(0) (Proposition 7). Since, *B*(0) is an ideal in each weak-BCC-algebra, from the last, we obtain (*x*∗*z* ^*m*−*n*^)∗*y* ∈ *B*(0), and consequently, *x*∗*z* ^*m*−*n*^ ∈ *B*(0). But, *x*∗*z* ^*m*−*n*^ ∈ *B*(*a*∗*c* ^*m*−*n*^), so *B*(0) = *B*(*a*∗*c* ^*m*−*n*^); that is, 0 = *a*∗*c* ^*m*−*n*^. This in the group (*I*(*G*); ·, ^−1^, 0) connected with *I*(*G*) gives 0 = *a* · *c* ^*n*−*m*^ = *a*. So, *x* ∈ *B*(0).

Now let *m* < *n*. Then (*x*∗*z* ^*m*^)∗(*y*∗*z* ^*n*^) ⩽ *x*∗(*y*∗*z* ^*n*−*m*^). This, similarly as in the previous case, for (*x*∗*z* ^*m*^)∗(*y*∗*z* ^*n*^) ∈ *B*(0) gives (*x*∗(*y*∗*z* ^*n*−*m*^))∗((*x*∗*z* ^*m*^)∗(*y*∗*z* ^*n*^)) ∈ *B*(0). Consequently, *x*∗(*y*∗*z* ^*n*−*m*^) ∈ *B*(0)∩*B*(*a*∗(0∗*c* ^*n*−*m*^)). So, 0 = *a*∗(0∗*c* ^*n*−*m*^). This in the group (*I*(*G*); ·,^−1^, 0) implies 0 = *a* · *c* ^*n*−*m*^ = *a*. Hence, *x* ∈ *B*(0).

The proof is complete.

The assumption on the number of elements of the set *I*(*G*) is essential; if *k* is not a divisor of |*m* − *n*|, then *B*(0) may not be an (*m*, *n*)-fold *p*-ideal.

**Example 40**

The solid weak-BCC-algebra *G* defined by

(29) ∗012345005015111015052250151334504244215055515010

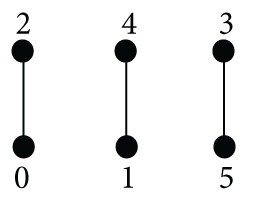
(30)
is proper, because (3∗1)∗4 ≠ (3∗4)∗1. The set *I*(*G*) has three elements. The set *B*(0) = {0,2} is an *n*-fold *p*-ideal for every natural *n* but it is not a (3,2)-fold ideal because (1∗1^3^)∗(0∗1^2^) ∈ *B*(0) and 1 ∉ *B*(0).

In the case when *B*(0) has only one element, the equivalence relation induced by *B*(0) has one-element equivalence classes. Since these equivalence classes are branches, a weak-BCC-algebra with this property is group-like. Direct computations show that in this case, *B*(0) is an *n*-fold *p*-ideal for every natural *n*.

This observation together with the just proved results suggests simple characterization of group-like weak-BCC-algebras.

**Theorem 41**

A weak-BCC-algebra *G* is group-like if and only if for some *n*⩾1 and all *x*, *z* ∈ *G*

(31) $$\begin{array}{c} \left(x*z^{n}\right)*\left(0*z^{n}\right)=0\Rightarrow x=0. \end{array}$$

**Proof**

Let *G* be a weak-group-like BCC-algebra. Then, *G* = *I*(*G*), which means that *G* has a discrete order; that is, *x* ⩽ *y* implies *x* = *y*. Since for *x*, *y*, *z* ∈ *G* we have (*x*∗*z* ^*n*^)∗(*y*∗*z* ^*n*^) ⩽ *x*∗*y*, a group-like weak-BCC-algebra satisfies the identity (*x*∗*z* ^*n*^)∗(*y*∗*z* ^*n*^) = *x*∗*y*. In particular, for *y* = 0, we have (*x*∗*z* ^*n*^)∗(0∗*z* ^*n*^) = *x*∗0 = *x*. So, (*x*∗*z* ^*n*^)∗(0∗*z* ^*n*^) = 0 implies *x* = 0.

Conversely, if the above implication is valid for all *x*, *z* ∈ *G*, then

(32) $$\begin{array}{c} 0=\left(x*z^{n}\right)*\left(0*z^{n}\right)⩽x*0=x \end{array}$$

gives 0 ⩽ *x*. This, according to the assumption, implies *x* = 0. Hence, *B*(0) = {0}, which means that *G* is group-like.

Remember that an ideal *A* of a weak-BCC-algebra is called *closed* if 0∗*x* ∈ *A* for every *x* ∈ *A*, that is, if *φ*(*A*) ⊂ *A*.

**Theorem 42**

For an (*m*, *n*)-fold *p*-ideal *A* of a solid weak-BCC-algebra *G*, the following statements are equivalent:

1. *A* is a closed (*m*, *n*)-fold *p*-ideal of *G*,
2. *I*(*A*) is a closed (*m*, *n*)-fold *p*-ideal of *I*(*G*),
3. *I*(*A*) is a subalgebra of *I*(*G*),
4. *A* is a subalgebra of *G*.

**Proof**

The implication (1)⇒(2) follows from Theorem 33.

(2)⇒(3) Observe first that *I*(*A*) is a closed BCK-ideal of *I*(*G*) and *a*∗*b* = *c* ∈ *I*(*G*) for any *a*, *b* ∈ *I*(*A*). Since *I*(*G*) is a group-like subalgebra of *G* (Corollary 11), in the group (*I*(*G*); ·, ^−1^, 0), we have *c* = *a* · *b* ^−1^ (Theorem 9), which means that *c* · *b* = *a* ∈ *I*(*A*). Thus,

(33) c·(0·b)=c∗(0·b)−1=a=c∗(b−1)−1=c∗b∈I(A).

Hence, *c*∗(0∗*b*) ∈ *I*(*A*). But 0∗*b* ∈ *I*(*A*) and *I*(*A*) is a BCK-ideal of *I*(*G*); therefore *c* ∈ *I*(*A*). Consequently, *a*∗*b* ∈ *I*(*A*) for every *a*, *b* ∈ *I*(*A*). So, *I*(*A*) is a subalgebra of *I*(*G*).

(3)⇒(4)  *I*(*A*) ⊂ *A*, so 0 ∈ *A*. Let *x* ∈ *B*(*a*), *y* ∈ *B*(*b*). If *x*, *y* ∈ *A*, then *a*, *b* ∈ *I*(*A*), and by the assumption *a*∗*b* ∈ *I*(*A*). From this, we obtain *x*∗*y* ∈ *B*(*a*)∗*B*(*b*) = *B*(*a*∗*b*), which together with Theorem 33 proves *x*∗*y* ∈ *A*. Hence, *A* is a subalgebra of *G*.

The implication (4)⇒(1) is obvious.

## 5. Nilpotent Weak-BCC-Algebras

A special role in weak-BCC-algebras play elements having a finite “order,” that is, elements for which there exists some natural *k* such that 0∗*x* ^*k*^ = 0. We characterize sets of such elements and prove that the properties of such elements can be described by the properties of initial elements of branches containing these elements.

**Definition 43**

An element *x* of a weak-BCC-algebra *G* is called *nilpotent*, if there exists some positive integer *k* such that 0∗*x* ^*k*^ = 0. The smallest *k* with this property is called the *nilpotency index* of *x* and is denoted by *n*(*x*). A weak-BCC-algebra in which all elements are nilpotent is called *nilpotent*.

By *N*_*k*_(*G*), we denote the set of all nilpotent elements *x* ∈ *G* such that *n*(*x*) = *k*. *N*(*G*) denotes the set of all nilpotent elements of *G*. It is clear that *N*_1_(*G*) = *B*(0).

**Example 44**

In the weak-BCC-algebras defined by (4) and (5), we have *n*(0) = *n*(1) = 1, *n*(2) = *n*(3) = 2.

**Example 45**

In the weak-BCC-algebra defined by

(34) ∗0abcde0000dcdaa0adcdbbb0dcdcccc0d0ddddc0ceecead0

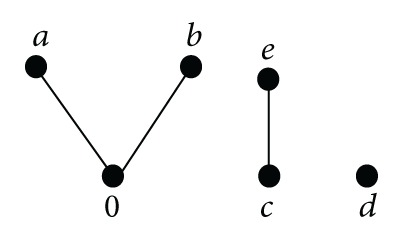
(35)
there are no elements with *n*(*x*) = 2, but there are three elements with *n*(*x*) = 3 and three with *n*(*x*) = 1.

**Proposition 46**

Elements belonging to the same branch have the same nilpotency index.

**Proof**

Let *x* ∈ *B*(*a*). Then *a* ⩽ *x*, which, by Theorem 3, implies 0∗*a* = 0∗*x*. This together with *a* ⩽ *x* gives 0∗*x* ^2^ ⩽ (0∗*a*)∗*x* ⩽ 0∗*a* ^2^. Hence, 0∗*x* ^2^ ⩽ 0∗*a* ^2^. In the same manner from 0∗*x* ^*k*^ ⩽ 0∗*a* ^*k*^, it follows that 0∗*x* ^*k*+1^ ⩽ 0∗*a* ^*k*+1^, which by induction proves 0∗*x* ^*m*^ ⩽ 0∗*a* ^*m*^ for every *x* ∈ *B*(*a*) and any natural *m*. Thus, 0∗*a* ^*m*^ = 0 implies 0∗*x* ^*m*^ = 0. On the other hand, from 0∗*x* ^*m*^ = 0, we obtain 0 ⩽ 0∗*a* ^*m*^. This implies 0 = 0∗*a* ^*m*^ since 0,0∗*a* ^*m*^ ∈ *I*(*G*) and elements of *I*(*G*) are incomparable. Therefore, 0∗*x* ^*m*^ = 0 if and only if 0∗*a* ^*m*^ = 0. So, *n*(*x*) = *n*(*a*) for every *x* ∈ *B*(*a*).

**Corollary 47**

A weak-BCC-algebra *G* is nilpotent if and only if its subalgebra *I*(*G*) is nilpotent.

**Corollary 48**

*x* ∈ *B*(*a*)∩*N*_*k*_(*G*)⇒*B*(*a*) ⊂ *N*_*k*_(*G*).

The above results show that the study of nilpotency of a given weak-BCC-algebras can be reduced to the study of nilpotency of its initial elements.

**Proposition 49**

Let *G* be a weak-BCC-algebra. If *I*(*G*) is a BCI-algebra, then *N*_*k*_(*G*) is a subalgebra and a BCC-ideal of *G* for every *k*.

**Proof**

Obviously, 0 ∈ *N*_*k*_(*G*) for every *k*. Let *x*, *y* ∈ *N*_*k*_(*G*). Then 0∗*x* ^*k*^ = 0∗*y* ^*k*^ = 0 and 0∗*x* ^*k*^ = 0∗*a* ^*k*^ = 0, 0∗*y* ^*k*^ = 0∗*b* ^*k*^ = 0 for some *a*, *b* ∈ *I*(*G*). Since *I*(*G*) is a BCI-algebra, by Theorem 16, we have 0 = (0∗*a* ^*k*^)∗(0∗*b* ^*k*^) = 0∗(*a*∗*b*)^*k*^. Hence, *a*∗*b* ∈ *N*_*k*_(*G*). Consequently, *x*∗*y* ∈ *B*(*a*)∗*B*(*b*) = *B*(*a*∗*b*) ⊂ *N*_*k*_(*G*). So, *N*_*k*_(*G*) is a subalgebra of *G*.

Now let *x* ∈ *B*(*a*), *y* ∈ *B*(*b*), *z* ∈ *B*(*c*). If *y*, (*x*∗*y*)∗*z* ∈ *N*_*k*_(*G*), then also *b*, (*a*∗*b*)∗*c* ∈ *N*_*k*_(*G*). Thus, 0∗*b* ^*k*^ = 0 and

(36) 0∗(a∗c)k=(0∗ak)∗(0∗ck)=((0∗ak)∗(0∗bk))∗(0∗ck)=0∗((a∗b)∗c)k=0,

which implies *a*∗*c* ∈ *N*_*k*_(*G*). This together with Corollary 48 implies *x*∗*z* ∈ *B*(*a*∗*c*) ⊂ *N*_*k*_(*G*). Therefore, *N*_*k*_(*G*) is a BCC-ideal of *G*. Clearly, it is a BCK-ideal, too.

**Corollary 50**

*N*_*k*_(*G*) is a subalgebra of each solid weak-BCC-algebra.

**Proposition 51**

*N*(*G*) is a subalgebra of each weak-BCC-algebra *G* in which *I*(*G*) is a BCI-algebra.

**Proof**

Since *N*(*G*) = ⋃_*k*∈*N*_ *N*_*k*_(*G*) and 0 ∈ *N*_*k*_(*G*) for every *k*, the set *N*(*G*) is nonempty. Let *x* ∈ *B*(*a*), *y* ∈ *B*(*b*). If *x*, *y* ∈ *N*(*G*) and *n*(*x*) = *m*, *n*(*y*) = *n*, then 0∗*x* ^*m*^ = 0∗*y* ^*n*^ = 0. From this, by Proposition 46, we obtain 0∗*a* ^*m*^ = 0∗*b* ^*n*^ = 0, which in the group (*I*(*G*); ·, ^−1^, 0) can be written in the form *a* ^−*m*^ = *b* ^−*n*^ = 0. But *I*(*G*) is a BCI-algebra; hence, (*I*(*G*); ·, ^−1^, 0) is an abelian group. Thus,

(37) 0=(a−m)n·(bn)m=a−mn·bmn=(a·b−1)−mn=0∗(a·b)mn,

by Theorem 9. Hence, *a*∗*b* ∈ *N*(*G*). This implies *x*∗*y* ∈ *B*(*a*∗*b*) ⊂ *N*(*G*). Therefore, *N*(*G*) is a subalgebra of *G*.

**Corollary 52**

*N*(*G*) is a subalgebra of each solid weak-BCC-algebra.

**Corollary 53**

Any solid weak-BCC-algebra *G* with finite *I*(*G*) is nilpotent.

**Proof**

Indeed, *I*(*G*) is a maximal group-like BCI-algebra contained in any solid weak-BCC-algebra. Hence, the group (*I*(*G*); ·, ^−1^, 0) is abelian. If it is finite, then each of its element has finite order *k*. Thus, 0∗*a* ^*k*^ = 0 · *a* ^−*k*^ = 0 for every *a* ∈ *I*(*G*). Consequently, *B*(*a*) ⊂ *N*_*k*_(*G*) ⊂ *N*(*G*) for every *a* ∈ *I*(*G*). Therefore, *G* = *N*(*G*).

**Corollary 54**

A solid weak-BCC-algebra *G* is nilpotent if and only if each element of the group (*I*(*G*); ·, ^−1^, 0) has finite order.

**Corollary 55**

In a solid weak-BCC-algebra *G*, the nilpotency index of each *x* ∈ *N*(*G*) is a divisor of *Card*(*I*(*G*)).

## 6. *k*-Nilradicals of Solid Weak-BCC-Algebras

The theory of radicals in BCI-algebras was considered by many mathematicians from China (cf. [18]). Obtained results show that this theory is almost parallel to the theory of radicals in rings. But results proved for radicals in BCI-algebras cannot be transferred to weak-BCC-algebras.

In this section, we characterize one analog of nilradicals in weak-BCC-algebras. Further, this characterization will be used to describe some ideals of solid weak-BCC-algebras.

We begin with the following definition.

**Definition 56**

Let *A* be a subset of solid weak-BCC-algebra *G*. For any positive integer *k* by a *k*-*nilradical of A*, denoted by [*A*; *k*], we mean the set of all elements of *G* such that 0∗*x* ^*k*^ ∈ *A*; that is,

(38) $$\begin{array}{c} \left[A;k\right]=\left\{x\in G:0*x^{k}\in A\right\}. \end{array}$$

**Example 57**

In the weak-BCC-algebra *G* defined in Example 44 for *A* = {0, *a*} and any natural *k*, we have [*A*; 3*k* + 1] = [*A*; 3*k* + 2] = *B*(0), [*A*; 3*k*] = *G*. But for *B* = {*a*, *e*}, we get [*B*; 3*k* + 1] = {*d*}, [*B*; 3*k* + 2] = *B*(*c*). The set [*B*; 3*k*] is empty.

**Example 58**

The solid weak-BCC-algebra *G* defined by

(39) ∗012345000005511022442200055332204444555025555500

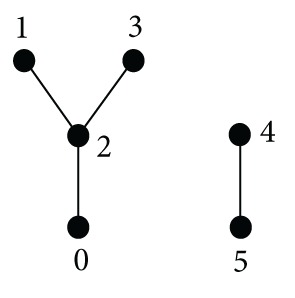
(40)
is proper, because (3∗4)∗5 ≠ (3∗5)∗4. In this algebra, each *k*-nilradical of *A* = {0,5} is equal to *G*; each *k*-nilradical of *B* = {1,4} is empty.

The first question is when for a given nonempty set *A* its *k*-nilradical is also nonempty? The answer is given in the following proposition.

**Proposition 59**

A *k*-nilradical [*A*; *k*] of a nonempty subset *A* of a weak-BCC-algebra *G* is nonempty if and only if *A* contains at least one element *a* ∈ *I*(*G*).

**Proof**

From the proof of Theorem 16, it follows that 0∗*x* ^*k*^ = 0∗*a* ^*k*^ for every *x* ∈ *B*(*a*) and any positive *k*. So, *x* ∈ [*A*; *k*] if and only if 0∗*a* ^*k*^ ∈ *A*. The last means that 0∗*a* ^*k*^ ∈ *A*∩*I*(*G*) because *I*(*G*) is a subalgebra of *G*.

**Corollary 60**

[*I*(*G*); *k*] = *G* for every *k*.

**Proof**

Indeed, 0∗*x* ^*k*^ = 0∗*a* ^*k*^ ∈ *I*(*G*) for every *x* ∈ *G*. Thus, *x* ∈ [*I*(*G*); *k*].

**Corollary 61**

If *I*(*G*) has *n* elements, then [*A*; *n*] = *G* for any subset *A* of *G* containing 0, and [*A*; *n*] = *∅* if 0 ∉ *A*.

**Proof**

Similarly, as in previous proofs, we have 0∗*x* ^*k*^ = 0∗*a* ^*k*^ for every *x* ∈ *B*(*a*) and any *k*. Since 0∗*a* ^*k*^ ∈ *I*(*G*) and *I*(*G*) is a group-like subalgebra of *G*, 0∗*x* ^*k*^ = *a* ^−*k*^ in the group (*I*(*G*); ·, ^−1^, 0) (Theorem 9). If *I*(*G*) has *n* elements, then obviously 0∗*x* ^*n*^ = *a* ^−*n*^ = 0 ∈ *A*. Hence, *x* ∈ [*A*; *n*]. This completes the proof.

**Corollary 62**

Let *x* ∈ *B*(*a*). Then *x* ∈ [*A*; *k*] if and only if *B*(*a*)⊂[*A*; *k*].

**Proof**

Since 0∗*x* ^*k*^ = 0∗*a* ^*k*^, we have *x* ∈ [*A*; *k*]⇔*a* ∈ [*A*; *k*].

**Corollary 63**

[*A*; *k*] = ⋃{*B*(*a*) : 0∗*a* ^*k*^ ∈ *A*}.

**Proposition 64**

Let *G* be a solid weak-BCC-algebra. Then for every positive integer *k* and any subalgebra *A* of *G* a *k*-nilradical [*A*; *k*] is a subalgebra of *G* such that *A*⊆[*A*; *k*].

**Proof**

Let *x*, *y* ∈ [*A*; *k*]. Then 0∗*x* ^*k*^, 0∗*y* ^*k*^ ∈ *A* and 0∗(*x*∗*y*)^*k*^ = (0∗*x* ^*k*^)∗(0∗*y* ^*k*^) ∈ *A*, by Theorem 16. Hence, *x*∗*y* ∈ [*A*; *k*]. Clearly *A*⊆[*A*; *k*].

**Proposition 65**

In a solid weak-BCC-algebra, a *k*-nilradical of an ideal is also an ideal.

**Proof**

Let *A* be a BCC-ideal of *G*. If *y* ∈ [*A*; *k*] and (*x*∗*y*)∗*z* ∈ [*A*; *k*], then 0∗*y* ^*k*^ ∈ *A* and *A*∋0∗((*x*∗*y*)∗*z*)^*k*^ = ((0∗*x* ^*k*^)∗(0∗*y* ^*k*^))∗(0∗*z* ^*k*^), by Theorem 16. Hence, *A*∋(0∗*x* ^*k*^)∗(0∗*z* ^*k*^) = 0∗(*x*∗*z*)^*k*^. Thus, *x*∗*z* ∈ [*A*; *k*].

Note that the last two propositions are not true for weak-BCC-algebras which are not solid.

**Example 66**

The weak-BCC-algebra *G* induced by the symmetric group *S*_3_ is not solid because *S*_3_ is not an abelian group (Corollary 14). Routine calculations show that *A* = {0,3} is a subalgebra and a BCC-ideal of this weak-BCC-algebra, but [*A*; 3] = {0,1, 2,3} is neither ideal nor subalgebra.

**Theorem 67**

In a solid weak-BCC-algebra, a *k*-nilradical of an (*m*, *n*)-fold *p*-ideal is also an (*m*, *n*)-fold *p*-ideal.

**Proof**

By Proposition 65, a *k*-nilradical of an (*m*, *n*)-fold *p*-ideal *A* of *G* is an ideal of *G*. If *y*, (*x*∗*z* ^*m*^)∗(*y*∗*z* ^*n*^)∈[*A*; *k*], then 0∗*y* ^*k*^, 0∗((*x*∗*z* ^*m*^)∗(*y*∗*z* ^*n*^)^*k*^) ∈ *A*. Hence, applying Theorem 16, we obtain

(41) ((0∗xk)∗((0∗zk)m))∗((0∗yk)∗((0∗zk)n)) =0∗((x∗zm)∗(y∗zn)k)∈A.

Thus, 0∗*x* ^*k*^ ∈ *A*. So, *x* ∈ [*A*; *k*].

Note that in general, a *k*-nilradical [*A*; *k*] of an ideal *A* does not save all properties of an ideal *A*. For example, if an ideal *A* is a horizontal ideal, that is, *x* ∈ *A*∩*B*(0)⇔*x* = 0, then a *k*-nilradical [*A*; *k*] may not be a horizontal ideal. Such situation takes place in a weak-BCC-algebra defined by (34). In this algebra, we have 0∗*x* ^3^ = 0 for all elements. Hence, *x* ∈ [*A*; 3]∩*B*(0) means that 0∗*x* ^3^ ∈ *A* and *x* ∈ *B*(0) which is also true for *x* ≠ 0.

Nevertheless, properties of many main types of ideals are saved by their *k*-nilradicals. Below, we present the list of the main types of ideals considered in BCI-algebras and weak-BCC-algebras.

**Definition 68**

An ideal *A* of a weak-BCC-algebra *G* is called

(i) *antigrouped*, if
  (42) $$\begin{array}{c} \varphi^{2}\left(x\right)\in A\Rightarrow x\in A, \end{array}$$
(ii) *associative*, if
  (43) $$\begin{array}{c} \left(x*y\right)*z,\;y*z\in A\Rightarrow x\in A, \end{array}$$
(iii) *quasiassociative* if
  (44) $$\begin{array}{c} x*\left(y*z\right),\;y\in A\Rightarrow x*z\in A, \end{array}$$
(iv) *closed*, if
  (45) $$\begin{array}{c} x\in A\Rightarrow 0*x\in A, \end{array}$$
(v) *commutative*, if
  (46) $$\begin{array}{c} x*y\in A\Rightarrow x*\left(y*\left(y*x\right)\right)\in A, \end{array}$$
(vi) *subcommutative*, if
  (47) $$\begin{array}{c} y*\left(y*\left(x*\left(x*y\right)\right)\right)=\in A\Rightarrow x*\left(x*y\right)\in A, \end{array}$$
(vii) *implicative* if
  (48) $$\begin{array}{c} \left(x*y\right)*z,\;y*z\in A\Rightarrow x*z\in A, \end{array}$$
(viii) *subimplicative* if
  (49) $$\begin{array}{c} \left(x*\left(x*y\right)\right)*\left(y*x\right)\in A\Rightarrow y*\left(y*x\right)\in A, \end{array}$$
(ix) *weakly implicative* if
  (50) $$\begin{array}{c} \left(x*\left(y*x\right)\right)*\left(0*\left(y*x\right)\right)\in A\Rightarrow x\in A, \end{array}$$
(x) *obstinate*, if
  (51) $$\begin{array}{c} x,y\notin A\Rightarrow x*y,\;y*x\in A, \end{array}$$
(xi) *regular*, if
  (52) $$\begin{array}{c} x*y,\;x\in A\Rightarrow y\in A, \end{array}$$
(xii) *strong*, if
  (53) $$\begin{array}{c} x\in A,\;y\in X-A\Rightarrow x*y\in X-A, \end{array}$$

for all *x*, *y*, *z* ∈ *G*.

**Definition 69**

We say that an ideal *A* of a weak-BCC-algebra *G* has the property *𝒫* if it is one of the above types, that is, if it satisfies one of implications mentioned in the above definition.

**Theorem 70**

If an ideal *A* of a solid weak-BCC-algebra *G* has the property *𝒫*, then its *k*-nilradical [*A*; *k*] also has this property.

**Proof**

(1)  *A* is antigrouped. Let *φ* ^2^(*x*)∈[*A*; *k*]. Then 0∗(*φ* ^2^(*x*))^*k*^ ∈ *A*. Since, by Theorem 3, *φ* ^2^ is an endomorphism of each weak-BCC-algebra, we have

(54) φ2(0∗xk)=φ2(0)∗(φ2(x))k=0∗(φ2(x))k∈A.

Thus, *φ* ^2^(0∗*x* ^*k*^) ∈ *A*, which according to the definition implies 0∗*x* ^*k*^ ∈ *A*. Hence, *x* ∈ [*A*; *k*].

(2)  *A* is associative. If (*x*∗*y*)∗*z*,  *y*∗*z* ∈ [*A*; *k*], then 0∗((*x*∗*y*)∗*z*)^*k*^ ∈ *A* and 0∗(*y*∗*z*)^*k*^ ∈ *A* which, in view of Theorem 16, means that ((0∗*x* ^*k*^)∗(0∗*y* ^*k*^))∗(0∗*z* ^*k*^) ∈ *A* and (0∗*y* ^*k*^)∗(0∗*z* ^*k*^) ∈ *A*. Since an ideal *A* is associative, this implies 0∗*x* ^*k*^ ∈ *A*; that is, *x* ∈ [*A*; *k*].

(3)  *A* is quasiassociative. Similarly as in the previous case *x*∗(*y*∗*z*),  *y* ∈ [*A*; *k*] means that 0∗(*x*∗(*y*∗*z*))^*k*^ ∈ *A* and 0∗*y* ^*k*^ ∈ *A*. Hence, (0∗*x* ^*k*^)∗((0∗*y* ^*k*^)∗(0∗*z* ^*k*^)) ∈ *A*. This implies 0∗(*x*∗*z*)^*k*^ = (0∗*x* ^*k*^)∗(0∗*z* ^*k*^) ∈ *A*. Consequently, *x*∗*z* ∈ [*A*; *k*].

(4)  *A* is closed. Let *x* ∈ [*A*; *k*]. Then, 0∗*x* ^*k*^ ∈ *A*. Thus,

(55) $$\begin{array}{c} 0*{\left(0*x\right)}^{k}=0*\left(0*x^{k}\right)\in A. \end{array}$$

So, 0∗*x* ∈ [*A*; *k*].

(5)  *A* is commutative. Let *x*∗*y* ∈ [*A*; *k*]. Then, 0∗(*x*∗*y*)^*k*^ ∈ *A*. From this, we obtain (0∗*x* ^*k*^)∗(0∗*y* ^*k*^) ∈ *A*, which gives 0∗(*x*∗(*y*∗(*y*∗*x*)))^*k*^ = (0∗*x* ^*k*^)∗((0∗*y* ^*k*^)∗((0∗*y* ^*k*^)∗(0∗*x* ^*k*^))) ∈ *A*. Hence, *x*∗(*y*∗(*y*∗*x*))∈[*A*; *k*].

For other types of ideals, the proof is very similar.
